# Illuminating immunotherapy response via precision T cell-targeted PET imaging

**DOI:** 10.3389/fmed.2024.1233913

**Published:** 2024-07-22

**Authors:** Sarah E. Glazer, Shivaani Kummar, Erik Mittra

**Affiliations:** ^1^Division of Internal Medicine, Oregon Health & Science University, Portland, OR, United States; ^2^Division of Hematology and Medical Oncology, Knight Cancer Institute, Oregon Health & Science University, Portland, OR, United States; ^3^Division of Molecular Imaging and Therapy, Oregon Health & Science University, Portland, OR, United States

**Keywords:** PET, immunotherapy, radiopharmaceuticals, tumor immune microenvironment, predictive biomarkers

## Abstract

Traditionally, immunotherapy agent selection and treatment strategies are guided by biopsy-based histological information. However, biopsies are limited in that they are invasive, provide static information regarding the tumor immune microenvironment, and only sample a small part of one tumor site. The tumor microenvironment is dynamic and heterogenous. As a result, the immune milieu at one site may be distinct from other metastatic sites. These factors make identifying which patients are likely to respond to different immunotherapies and which harbor intrinsic resistance mechanisms difficult to identify based on a biopsy alone. As such, there is significant interest in alternative methodologies that better characterize the tumor immune microenvironment and monitor immunotherapy response. PET imaging potentially offers a non-invasive way to characterize the tumor immune microenvironment at the primary tumor and metastases and allow for longitudinal characterization. Herein, we review pre-clinically and clinically tested T cell-targeted PET radiopharmaceuticals, as T cells have been the dominant immunotherapy target, and their utility in both evaluating response to immunotherapy and in understanding the systemic immune response to treatment with immunotherapeutics.

## 1 Introduction

Therapeutic agents that modulate the immune system response to cancer cells have led to a paradigm shift in cancer therapy. Since the approval of anti-CTLA-4 for the treatment of metastatic melanoma in 2011, various T cell modulators, immune check point inhibitors (ICIs), CAR-T cells, TIL therapy, oncolytic viruses, and intra-tumor immune stimulators have emerged as promising therapeutics (1, 2). The mainstay of immunotherapy treatment has been the use of ICIs to modulate T cell inhibitory signals specifically focused on targeting CTLA-4 and the PD-1/PD-L1 axis (3). However, only approximately 20–40% of patients currently respond to ICI therapy (4). Additionally, many patients experience immune-related adverse effects (irAEs) (5). Thus, developing predictive biomarkers indicative of ICI response is imperative to effectively stratify patients into treatment groups.

Currently, there are three FDA-approved predictive biomarkers of ICI response: PD-L1 expression by IHC, microsatellite instability/defective mismatch repair (MSI/dMMR) either by immunohistochemistry (IHC), polymerase chain reaction (PCR), or next-generation sequencing (NGS), and tumor mutational burden (TMB) by whole exome sequencing (WES) or NGS (6). Although, overall higher PD-L1 expression by histology correlates with better response to anti-PD-L1 therapy, multiple trials have demonstrated that some tumors that are deemed PD-L1 negative by IHC still have positive responses to immunotherapy (7–10). Variability in staining between IHC anti-PD-L1 antibodies, the difference in IHC scoring criteria, and the dynamic changes in PD-L1 protein expression in response to therapy all contribute to the limitations in the clinical utility of this biomarker (11–14).

Biomarker assays that detect deficits in MSI/dMMR (microsatellite instability or mismatch repair proteins) proteins also have limitations. Cancers that have deficits in MSI/dMMR are likely to be immunotherapy responsive, but some cancer types that are responsive to ICIs, such as non-small cell lung cancer (NSCLC), have a very low frequency of MSI compared to other solid tumors such as colorectal cancer or gynecological cancers (15). Although higher overall tumor mutational burden (TMB) is predictive of better response to ICIs, there are no clear TMB-specific cutoffs that distinguish between ICI responders and non-responders. Similar to the predictive value of PD-L1 expression by histology, some tumors with low TMB still show robust responses to ICIs (16–18). Attempts have been made to set TMB cutoffs, such as the recent FDA approval of 10 mut/Mb as the cutoff for high TMB tumors and subsequent indication for treatment with Pembrolizumab (19). However, there remain concerns that a strict cutoff will exclude patients who may benefit from ICI therapy (20).

Many of these immunotherapy biomarkers rely on single static tissue samples ascertained from a small area of one tumor site. This type of profiling undervalues the dynamic nature of the tumor immune microenvironment and tumor heterogeneity at the primary and metastatic sites and is unable to capture systemic changes in real-time that occur in response to immunotherapy treatment. In addition, most predictive biomarkers have focused on the T cell inhibitor signals PD-1 and PD-L1. However, additional T cell modulatory proteins, as well as other immune cells, have been shown to play a role in generating or inhibiting an anti-cancer immune response. Novel T cell modulatory targets and immune-oncology agents targeting other cells in the tumor immune microenvironment, such as macrophages, myeloid cells, and NK cells, are currently under development. Positron emission tomography (PET) imaging is a non-invasive quantitative methodology that provides a whole-body evaluation of various aspects of tumor biology and can monitor changes in response to therapy at multiple time points throughout treatment. In addition, PET probes can be tailored to assess the spatial and temporal dynamics of a variety of proteins and immune cells present in the tumor microenvironment. In this review, we will focus on the strategies and effectiveness of current T cell-targeted PET radiopharmaceuticals that have been evaluated pre-clinically and clinically and their utility in monitoring systemic response to immunotherapy.

## 2 PET as a method for monitoring immunotherapy response

PET is a highly sensitive imaging modality in which radiolabeled compounds, peptides, antibodies, and antibody fragments are administered in the radiopharmaceutical (picomolar or nanomolar concentrations) regime and thereby provide a precision readout of a molecular target of interest without perturbing the molecular dynamics of the interrogated biological system (21). ^18^F-fluorodeoxyglucose (FDG) is the most common radiopharmaceutical in oncology. However, ^18^F-FDG, a marker of glucose utilization or metabolic activity, is unable to distinguish between cancer or immune cells in the tumor immune microenvironment (22). In addition, the infiltrating immune wave in response to ICI treatment can lead to pseudo-tumor progression (a positive marker of ICI response), which can be difficult to distinguish from actual tumor progression by traditional imaging criteria (23). This has led to updated iRECIST criteria to help account for immune-related response dynamics (24). Ultimately, imaging with more specific PET radiopharmaceuticals has the potential to untangle these immune-tumor dynamics, both within the tumor microenvironment and systemically, in response to ICI treatment (25–27).

Many PET radiopharmaceuticals have been developed to monitor and predict immunotherapy treatment response. Antibodies, antibody fragments, peptides, and small compounds have all been utilized to develop immunotherapy monitoring imaging agents. Radiopharmaceutical dynamics rely on the pharmacokinetics of the radiolabeled agents and the choice of radioisotope. The most common PET radioisotopes include zirconium-89, copper-64, gallium-68, and fluorine-18, each with a different half-life (zirconium-89 T_1/2_ = 78.4 h, copper-64 T_1/2_ = 12.7 h, gallium-68 T_1/2_ = 1.1 h, fluorine-18 T_1/2_ = 109.8 min) (28). Radioisotope and vector pairs are often chosen in such a way that the radioisotope half-life aligns with the vector pharmacokinetics.

Full-length antibodies are the most used imaging vectors for T cell imaging, specifically IgG class immunoglobulins with an epitope designed to target a protein of interest. Antibodies are 150 kD in size and have a slower blood clearance time (on the order of days) due to Fc receptor binding and re-circulation. As such, antibodies are often paired with isotopes with longer half-lives, such as zirconium-89. Due to the longer overall pharmacokinetics of antibody-based vectors, images are acquired 3–4 days after initial injection. These properties offer the advantage of multiday longitudinal imaging due to longer clearance times. However, antibody-based vectors are limited by poor tumor penetration due to their size as well as poorer tumor-to-background ratios due to off-target binding to high FcγR-expressing cells, such as immune cells in the spleen, which can act as an antibody sink. Off-target binding is often overcome with pre-treatment with cold (unlabeled) antibodies to saturate off-target FcγR expressing cells and increase tumor-to-background ratios. Small antibody fragment-based vectors, which do not include the Fc end, help overcome these challenges and benefit from faster blood clearance, better tumor penetration, and the ability to conduct same-day imaging. Antibody fragments are often paired with shorter half-life radioisotopes such as copper-64 and gallium-68. Finally, small molecule-based vectors have the fastest clearance time, which also allows for same-day imaging (28, 29).

Radiotracer uptake by PET is assessed either visually or by calculating standardized uptake values (SUVs). SUVs are calculated by determining the amount of radioactivity detected divided by an area or region of interest (ROI). There are no specific SUV cutoff values as there is large variability in overall biodistribution per patient. This variability is best accounted for by normalizing the lesion uptake to background areas such as the blood pool, liver, or adjacent soft-tissue (30). This often requires adjusting the thresholding either visually or numerically, but the best approach to interpretation varies by radiopharmaceutical. In addition, new strategies are being developed to help improve the accuracy of SUV measurements (30).

## 3 Radiopharmaceuticals that utilize or evaluate therapeutic antibody targets

### 3.1 PD-L1/PD-1

**Figure 1:**
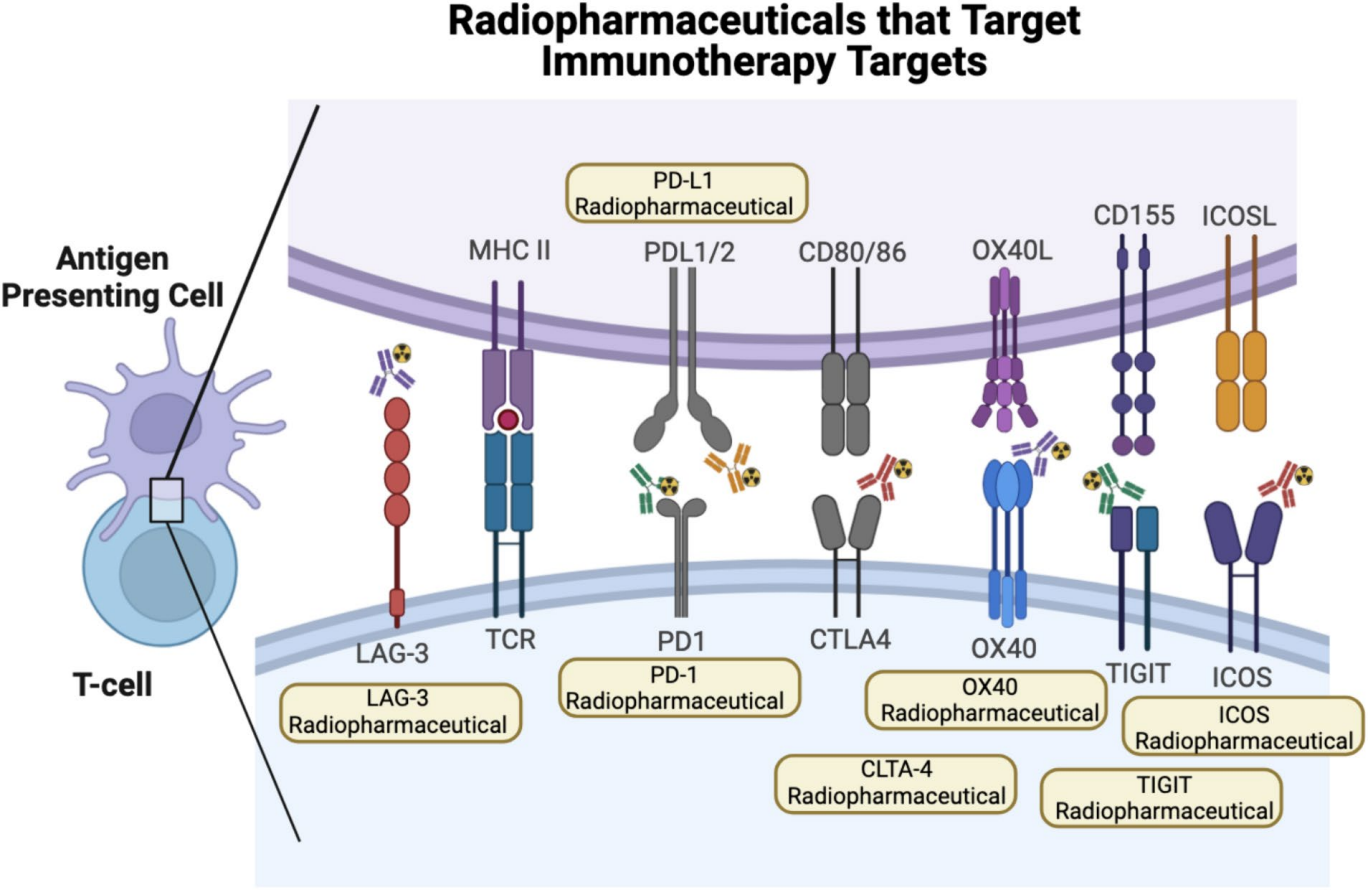
Radiopharmaceutical that target immunotherapy targets. One strategy to monitor ICI response has been to develop radiopharmaceuticals that monitor different immunotherapy targets, such as PD-1, PD-L1, CTLA-4, OX40, TIGIT, ICOS, and LAG-3. Created using Biorender.com.

Activation of T cells requires engagement of the T cell receptor (TCR) with antigens presented by MHCII on antigen-presenting cells (APCs). Engagement of PD-1, which is mainly expressed on exhausted CD8 T cells, with PD-L1, which is expressed in tumor tissue, DCs, macrophages, lymphocytes, stroma, and endothelial cells, leads to T cell inactivation (31) (Figure 1). The majority of clinically approved ICIs have focused on targeting the PD-L1/PD-1 axis, including the anti-PD-1 antibodies Nivolumab, Pembrolizumab, and Cemiplimab and the anti-PD-L1-targeted antibodies Durvalumab, Atezolizumab, and Avelumab (31). Several of these antibodies have been radiolabeled and evaluated clinically as potential biomarkers of immunotherapy response.

A variety of anti-PD-L1 and anti-PD-1 radiolabeled antibodies, pro-bodies, adnectins, and small molecules have been evaluated as potential predictors of response to immunotherapy in clinical trials, of which some were able to significantly predict response to therapy (32). ^89^Zr-Atezolizumab (anti-PD-L1) was investigated in 22 patients with metastatic and advanced non-small cell lung cancer (NSCLC), triple-negative breast cancer (TNBC), and bladder cancer (33). On a per-patient basis, high radiopharmaceutical uptake at tumor sites overall was indicative of response to therapy as evaluated by the iRECIST response criteria. In addition, higher initial tumor uptake, as measured by SUVmax, was significantly correlated with a change in lesion size in response to treatment. In addition, the calculated mean SUVmax was utilized to significantly stratify patients with longer overall progression-free and overall survival. Notably, radiotracer uptake did not correlate with PD-L1 tissue staining by IHC (33). Similarly, ^89^Zr-Pembrolizumab (anti-PD-1) was evaluated in two studies, one with 18 patients (11 with melanoma and 7 with NSCLC) and one that followed 12 patients with NSCLC. The first study showed that higher radiopharmaceutical uptake in tumors, as calculated by the SUVmax, was correlated with significant tumor response to therapy as well as longer overall progression-free and overall survival (34). Although the second study demonstrated that increased radiopharmaceutical uptake was significantly correlated with overall response as measured by the RECIST 1.1 criteria, higher uptake was only correlated, not statistically significant, with increased overall survival (35). Both studies also noted that radiopharmaceutical uptake was not correlated with IHC staining of PD-1 and PD-L1 (34). An evaluation of ^18^F-BMS-986192 (anti-PD-1 adnectin) and ^89^Zr-Nivolumab (anti-PD-1 antibody) in 13 patients with NSCLC treated with nivolumab demonstrated that significantly higher radiopharmaceutical tumor uptake, as well as higher uptake in tumors that were positive for PD-1 and PD-L1 by IHC, was significantly correlated with response to treatment. Notably, only staining of PD-1 by histology was significantly correlated with overall tumor response (36). In a second study evaluating ^18^F-BMS-986192 in 10 patients with metastatic melanoma, they were able to discriminate, by ROC analysis, tumors that were increasing or decreasing in size in response to ICI therapy by normalizing the radiopharmaceutical uptake in the tumor to the blood pool (37).

While several anti-PD-1 and anti-PD-L1 radiopharmaceuticals were able to predict response to immunotherapy in small clinical cohorts, evaluation of ^89^Zr-Durvalumab (anti-PD-L1) in 10 patients with NSCLC, ^89^Zr-Cx-072 (a PD-L1 pro-body) in 8 patients with a variety of tumor types, and ^68^Ga-WL12 (a peptide-based anit-PD-L1 agent) in 8 patients with NSCLC all demonstrated a correlation between radiopharmaceutical uptake and ICI response but these results were not statistically significant (38). Overall, PD-L1 and PD-1 targeted radiopharmaceuticals have demonstrated that they can be utilized to predict response to ICI therapy in smaller clinical studies. Although not all radiopharmaceuticals demonstrated statistically significant results, some of these studies were quite small and may not have been powered to demonstrate significant effects (Table 1).

**Table 1:** Summary of pre-clinical and clinical radiopharmaceuticals.

Name	Target	Label	Type	Stage
^89^Zr-Atezolizumab*	PD-L1	^89^Zr	Antibody	First in Human
^89^Zr-Durvalumab	PD-L1	^89^Zr	Antibody	Phase 1/Phase 2 Clinical Trial
^89^Zr-Cx-072	PD-L1	^89^Zr	Probody	First in Human
^89^Zr-Pembrolizumab*	PD-1	^89^Zr	Antibody	Phase 2 Clinical Trial
^18^F-BMS-986192*	PD-1	^18^F	adnectin	Phase 1 Clinical Trial
^89^Zr-Nivolumab*	PD-1	^89^Zr	Antibody	First in Human
^18^F-NOTA-F12	PD-1	^18^F	D-peptide	First in Human
^68^GA-WL12	PD-L1	^68^Ga	Peptide	First in Human
^64^Cu-DOTA-anti-CTLA-4	CTLA-4	^64^Cu	Antibody	Pre-clinical
^64^Cu-DOTA-ipilimumab	CTLA-4	^64^Cu	Antibody	Pre-clinical
^64^Cu-NOTA-ipilimumab- F(ab^’^)_2_	CTLA-4	^64^Cu	F(ab^’^)_2_ fragment	Pre-clinical
^89^Zr-BI754111	LAG-3	^89^Zr	Antibody	First in Human
^64^Cu-DOTA-AbOX40*	OX40	^64^Cu	Antibody	Pre-clinical
^89^Zr-DFO-OX40*	OX40	^89^Zr	Antibody	Pre-clinical
^89^Zr-DFO-ICOS*	ICOS	^89^Zr	Antibody	Pre-clinical
^89^Zr-TIGITmAb	TIGIT	^89^Zr	Antibody	Pre-clinical
^64^Cu-TIGITmAb	TIGIT	^64^Cu	Antibody	Pre-clinical
^68^Ga-GP12	TIGIT	^68^Ga	D-Peptide	Pre-clinical
^89^Zr-DFO-CD3	CD3	^89^Zr	antibody	Pre-clinical
^64^Cu-CD4-Nb1	CD4	^64^Cu	nanobody	Pre-clinical
^89^Zr-DFO-CD4*	CD4	^89^Zr	F(ab^’^)_2_ fragment	Pre-clinical
^64^Cu-NOTA-2.43 Mb	CD8	^64^Cu	antibody fragment	Pre-clinical
^89^Zr-malDFO-169 cDb	CD8	^89^Zr	cys-diabody	Pre-clinical
^89^Zr-IAB22M2	CD8	^89^Zr	minibody	Phase I Clinical Trial
^89^ZED88082A*	CD8	^89^Zr	one armed minibody	First in Human
^68^Ga-NOTA-GZP*	Granzyme-B	^68^Ga	cleavable peptide	Pre-clinical
[^18^F]AlF-mNOTA-GZP*	Granzyme-B	^18^F	cleavable peptide	Pre-clinical
^68^Ga-grazytracer*	Granzyme-B	^68^Ga	cleavable peptide	Pre-clinical*, First in Human
[^18^F]FB-IL2	IL-2	^18^F	peptide	First in Human
^89^Zr-anti-IFNγ*	IFNy	^89^Zr	antibody	Pre-clinical
[^18^F]F-AraG*	9-β-D-Arabinofuranosylguanine	^18^F	nucleoside	Pre-clinical*, Phase I and Phase II Clinical Trials
^18^F-FLT	3′-deoxy-3′-fluorothymidine	^18^F	Compound	First in Human

*Denotes that the radiopharmaceutical was effective in predicting response to immunotherapy and the subsequent study type, if there are multiple, is also denoted.

### 3.2 CTLA-4

Radiopharmaceuticals have also been developed to evaluate other immune-check point inhibitors and their utility in monitoring response to immunotherapy. CTLA-4, found on T cells, including CD4 helper T cells and T regs, competes with CD28 and binds CD80 or CD86 on antigen-presenting cells to confer T cell inhibitory signals (Figure 1). CTLA-4 expression has also been found on tumor tissue. The therapeutic anti-CTLA-4 antibody, ipilimumab, initially had success in treating melanoma and has now been approved for the treatment of multiple cancers. Pre-clinically, Higashikawa et al. labeled a murine CTLA-4 antibody with Cu64, ^64^Cu-DOTA-anti-CTLA-4, and evaluated its targeting in the CT26 tumor model, a syngeneic colon cancer model. They demonstrated increased tumor-specific uptake of ^64^Cu-DOTA-anti-CTLA-4, compared to tumor uptake of Cu64 labeled isotype control, demonstrating specificity (39). Ehldering et al. radiolabeled ipilimumab, ^64^Cu-DOTA-anti-CTLA-4, and evaluated this radiopharmaceutical in several lung cancer xenograft models, A549, H460, and H358. They demonstrated tumor-specific radiopharmaceutical accumulation with increased uptake in tumors with higher CTLA-4 expression. They also demonstrated specificity, as pre-injection with cold antibody diminished tumor-specific uptake (40). Ehldering et al. compared radiolabeled ipilimumab, ^64^Cu-NOTA-ipilimumab, to a radiolabeled ipilimumab F(ab^’^)_2_ fragment, ^64^Cu-NOTA-ipilimumab- F(ab^’^)_2_. Using these two radiopharmaceuticals, they monitored immune infiltration in humanized mice using a model of graft vs. host disease (GvHD). They showed higher specific uptake in the salivary glands, known to harbor CD3+ CTLA-4+ cells in humanized mice compared to immunocompromised mice. In addition, although the full antibody radiopharmaceutical demonstrated higher overall uptake in the salivary glands, the F(ab^’^)_2_ demonstrated better salivary gland-to-blood ratios and cleared faster (41). These pre-clinical studies demonstrate that PET radiopharmaceuticals can be utilized to detect CTLA-4 expressing immune and tumor cells *in vivo*; however, further work is needed to determine if this radiopharmaceutical may be an effective agent to monitor response to immunotherapy clinically.

### 3.3 Lag-3

LAG-3, which can be found in NK cells, T cells, macrophages, and dendritic cells, interacts with MHCII, leading to T cell inhibition (Figure 1). Anti-LAG-3 therapeutics have demonstrated limited therapeutic response as a monotherapy treatment but have shown some clinical efficacy when used in combination with other immunotherapy agents (42). Miedem et al. investigated a Zirconium-89 labeled anti-LAG-3 antibody, ^89^Zr-BI754111, in 2 patients with HNSCC and 4 patients with NSCLC who had already been treated with PD-1 or PD-L1 therapies. Patient received treatment with ezabenlimab (anti-PD-1) and 8 days later was imaged with ^89^Zr-BI754111. Patients were also imaged with FDG at baseline. ^89^Zr-BI754111 demonstrated uptake in all lesions also identified by FDG. Tumor uptake with ^89^Zr-BI754111 was overall heterogenous. Tumors with higher uptake were later confirmed to have higher LAG-3 and T cell infiltration by histology and RNA sequencing. In addition, patients with higher ^89^Zr-BI754111 uptake had slower overall growth rates in response to treatment, although these results were only correlative and not statistically significant. These results demonstrate that imaging of LAG-3 may be another methodology to measure potential response to immunotherapy but requires further review in larger clinical trials (43).

### 3.4 OX40

OX40 is expressed mainly on CD4 T cells, both activated effector T cells and regulatory T cells (44). Interaction of OX40 with OX40 ligand on T cells leads to proliferation and activation of T cells (Figure 1). OX40 is actively being explored as a new immune checkpoint agonist therapy in clinical trials (45). As such, OX40 radiopharmaceuticals may be able to evaluate systemic activation of T cells. Pre-clinically, Alam et al. radiolabeled a murine OX40 antibody with copper-64, ^64^Cu-DOTA-OX40. They utilized this radiopharmaceutical to monitor T cell infiltrated in mice harboring A20 tumors, a murine lymphoma model, that were injected with CpG, a TLR9 agonist that can lead to immune activation. Before intra-tumor CpG injection, ^64^Cu-DOTA-OX40 imaging demonstrated uptake in tumor-draining lymph nodes of tumor-bearing mice. Subsequently, post intra-tumoral CpG injection, they showed significant radiopharmaceutical uptake in the tumors and tumor-associated lymph nodes of treated mice. Flow cytometry analysis of the tumor confirmed that treated tumors had an increase in CD3 + OX40+ T cells. At later time points post-treatment, they also observed increased radiopharmaceutical uptake at distant tumor sites. Finally, initial tumor radiopharmaceutical uptake was successfully used to classify treated tumors into potential responders and non-responders to CpG and cancer vaccine therapy (46). In another pre-clinical study, Nobashi et al. investigated another murine OX40 radiopharmaceutical, ^89^Zr-DFO-OX40, and its utility in tracking T cell activation and dynamics. In this study, mice harboring orthotopic gliomas were treated with CpG-ODN, glioma lysates, and OX40 antibody treatment and then subsequently underwent imaging with ^89^Zr-DFO-OX40. Treated mice showed radiopharmaceutical uptake in tumor-draining lymph nodes, and lymph nodes were more distal from the tumor treatment site. In addition, lymph node radiopharmaceutical uptake was delayed and occurred a few days after treatment. ^89^Zr-DFO-OX40 uptake in the treated lymph nodes significantly correlated with response to treatment (47). These pre-clinical studies demonstrate that OX40-based radiopharmaceuticals have the potential to monitor the dynamics of T cell activation and antigen presentation systemically in response to an immunotherapy treatment but have yet to be tested clinically.

### 3.5 TIGIT

TIGIT is another promising new immunotherapy. TIGIT is expressed on T cells, NK cells, and Tregs and sends inhibitory signals to antigen-presenting cells (Figure 1). In its evaluation as a new potential immunotherapeutic target, targeted TIGIT therapeutics have shown the most promise in clinical trials when used in combination with anti-PD-1 therapy (48, 49). Pre-clinically, Shaffer et al. evaluated both Copper-64 labeled and Zirconium-89 labeled murine anti-TIGIT antibodies. They demonstrated uptake of both radiopharmaceuticals in xenograft HeLa models, with overall better performance of the Zirconium-89 labeled antibody. They were also able to demonstrate specificity with *in vivo* cold-blocking experiments (50). Wang et al. evaluated a Gallium-68 labeled D-peptide TIGIT antagonist ^68^Ga-GP12. Pre-clinically, they demonstrated that ^68^Ga-GP12 PET imaging showed tumor-specific uptake in mice harboring B16F10, Panc02, and MC38 tumors. Subsequently, by flow cytometry, they demonstrated that this uptake was localized to TIGIT expression on CD8 T cells, Tregs, and NK cells. Clinically, a pilot study of two patients with bronchogenic adenocarcinoma demonstrated ^68^Ga-GP12 uptake in the primary tumors and metastatic sites, as well as heterogenous uptake in the primary tumor (51). Weng et al. also radiolabeled a TIGIT D-peptide TBP-3 with Gallium-68. Pre-clinically, they were able to demonstrate tumor-specific uptake in mice harboring 4 T1 tumors known to express TIGIT (52). Although TIGIT-specific radiopharmaceuticals have yet to be utilized to monitor or predict immunotherapy response clinically, they offer a new avenue to monitor T cell and NK cell-specific tumor infiltration and dynamics.

### 3.6 ICOS

ICOS is a T cell co-stimulatory molecule that is expressed on CD4 and CD8 T cells following T cell receptor engagement. In addition, ICOS expression is also found on Treg cells, and thus, immunotherapy targets have been evaluated as potentiators of T cell signaling enhancement in combination with PD-L1/PD-1 and CTLA-4 targeting as well as a mechanism to reduce Treg cells that may impinge upon T cell proliferation (53). Because ICOS is induced on activated T cells, PET imaging agents that target ICOS could provide a readout of T cell activation in response to immunotherapy treatment or causes of T cell activation, such as in graft vs. host disease. To this end, in a pre-clinical study of GvHD, ^89^Zr-DFO-ICOS was utilized to monitor early T cell activation in a mouse model as a predictor of the disease. Radiopharmaceutical uptake in the lymph nodes, spleen, and intestines was able to distinguish, by PCA analysis, mice with GvHD and has the potential to be used as a predictive biomarker of early GvHD onset (54). In another pre-clinical study, ^89^Zr-DFO-ICOS was utilized to predict response to treatment with STING or PD-1 therapy in mice harboring Lewis lung cancer. They demonstrated radiopharmaceutical uptake both at the tumor and tumor-draining lymph node after initial treatment. They also found that ^89^Zr-DFO-ICOS uptake at both these sites initially after treatment was predictive of response to treatment subsequent days later (55). Although limited to pre-clinical studies, these models demonstrate that ICOS PET imaging agents have the potential to monitor early T cell activation as a surrogate marker for immunotherapy response.

PD-1- and PD-L1-targeted PET agents are the farthest along in clinical testing and have shown the most success among the PET agents designed to monitor immunotherapy targets in terms of utility in predicting response to treatment to immunotherapy. Although not all PD-1/PD-L1 PET agents showed statistically significant immunotherapy response predictive effects, many of these studies were smaller in size and likely not effectively powered to ascertain these differences. Notably, although higher radiopharmaceutical uptake at tumors was often predictive of response to immunotherapy, radiopharmaceutical uptake was often not correlated with PD-1 or PD-L1 expression by IHC. In addition, PD-1 and PD-L1 expression by IHC was also not significantly correlated with response to ICI in all studies. This discordance further highlights the challenges of PD-1/PD-L1 IHC as a predictive biomarker. IHC continues to have drawbacks in that it can only assess a small part of the tumor, whereas PET imaging allows for a whole-body readout and can readily evaluate the heterogeneity of multiple targets across multiple tumor sites. Among pre-clinical studies of immunotherapy target-based PET imaging agents, OX40 and ICOS were shown to be the most effective in predicting response to immunotherapy treatment. Notably, OX40 and ICOS are both markers of T cell activation; however, they have not been as extensively studied as PD-1/PD-L1 targeting PET imaging agents. Nevertheless, these early studies of imaging agents that monitor T cell activating signals highlight that in addition to being able to ascertain if the immunotherapy target is available, activation of the effector cells, in this case, T cells, may also be a key variable in predicting response to therapy.

## 4 T cell imaging

Activating an anti-cancer immune-mediated response is a multistep process, with blocking T cell inhibitory signals being only one part of the cascade. For immunotherapy to be effective, T cells must also be present in the tumor micro-environment and confer anti-tumor activity once T cell inhibitor signals are abrogated. Thus, monitoring T cell tumor infiltration and systemic activation is a potential strategy to monitor response to immunotherapy. One methodology to monitor T cell infiltration is to design radiopharmaceuticals targeted at T cell lineage proteins, such as CD3, CD4, and CD8.

### 4.1 Imaging of lineage markers

#### 4.1.1 CD3 radiopharmaceuticals

Some groups have focused on developing radiopharmaceuticals targeting CD3, a broadly expressed T cell lineage marker found both on CD8 and CD4 T cells. Vera et al. investigated a radiolabeled murine anti-CD3 radiopharmaceutical pre-clinically, ^89^Zr-DFO-CD3. In mice bearing BBN975 tumors, a syngeneic murine bladder cancer model, they demonstrated increased uptake of their radiopharmaceutical in the spleen, thymus, and tumor compared to labeled isotype control, demonstrating that a CD3 targeted radiopharmaceutical could be used to monitor tumor-specific T cell infiltration (56). Larimer et al. evaluated ^89^Zr-DFO-CD3, a radiolabeled anti-CD3 radiopharmaceutical in mice bearing CT26 tumors, an immunocompetent colon cancer model, that had been treated with anti-CTLA-4. They observed a bimodal distribution in tumors with high and low uptake, but this distribution was not statistically significant. Tumors that had initial high uptake of the CD3 radiopharmaceutical later had reduced tumor volume, with the authors concluding that higher initial radiopharmaceutical uptake was indicative of more T cell infiltration and heralded potential anti-CTLA-4 mediated ICI response (57). However, CD3 is a non-specific T cell marker as it is expressed on cytotoxic T cells, helper T cells, and Tregs. This lack of specificity can make CD3 monitoring a challenge; it will globally assess T cell infiltration, but it will be unable to distinguish between a pro-tumor T cell microenvironment, that is, Treg predominant, and an anti-tumor T cell microenvironment, that is, cytotoxic T cell predominant.

#### 4.1.2 CD4 radiopharmaceuticals

**Figure 2:**
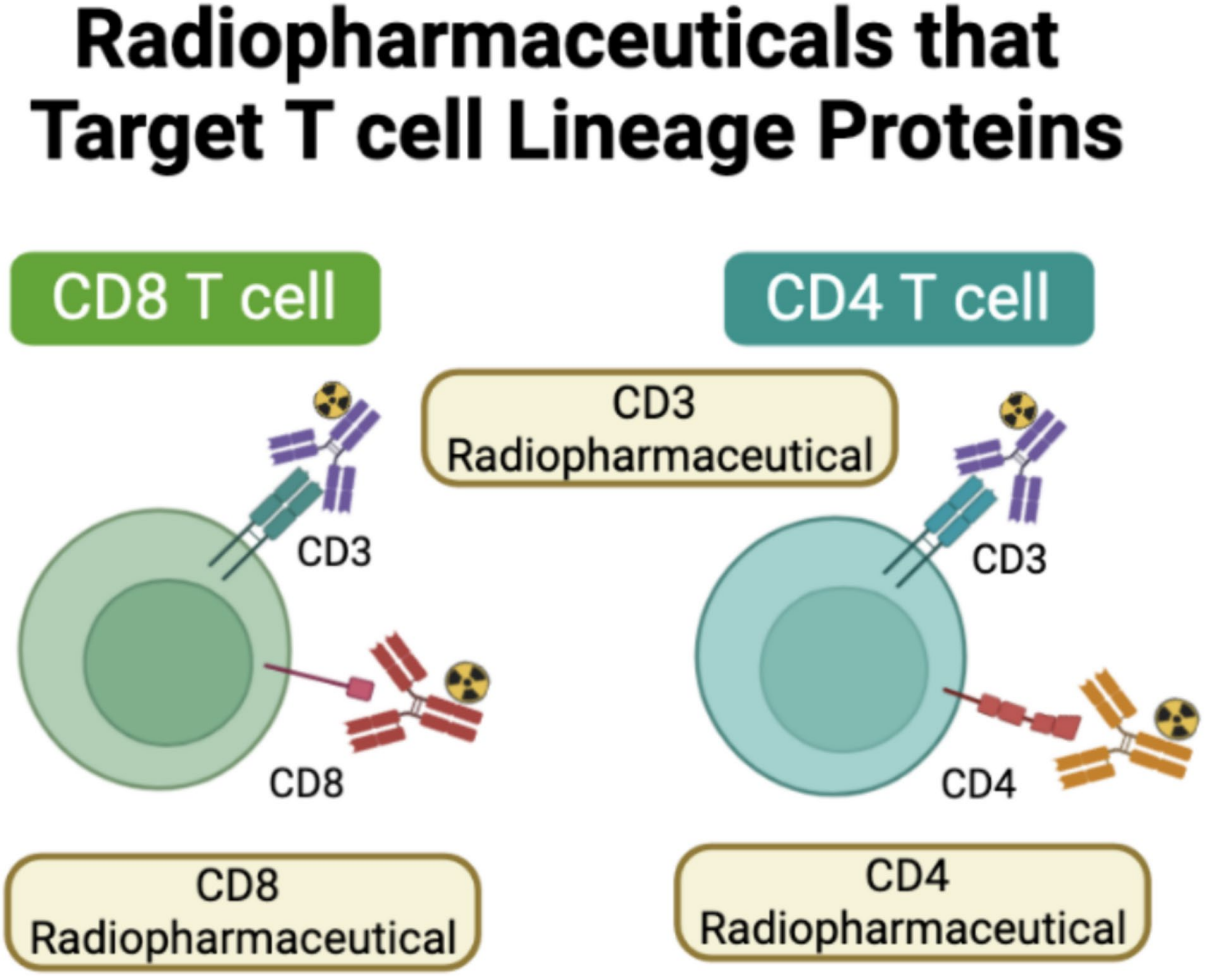
Radiopharmaceuticals that target T cell lineage proteins. Another strategy has been to develop radiopharmaceuticals directly targeted at T cell lineage proteins, such as CD3, CD4, and CD8. Created using Biorender.com.

CD4 T cells, once stimulated, generate helper T cells as well as Tregs. Tregs can dampen T cell effector function, whereas CD4 helper T cells play a critical role in the development of anti-tumor immunity by assisting in the development of cytotoxic CD8 T cells, secreting anti-tumor cytokines such as IFNγ and TNFα, and stimulating B cell production (58) (Figure 2). Thus, radiopharmaceuticals specific to CD4 may offer a more precise avenue to monitor tumor-specific T cell infiltration in real-time. Traenkle et al. developed several human-specific CD4 nanobodies. When these nanobodies were radiolabeled with Copper-64, they were able to demonstrate CD4-specific uptake in a human CD4 knock-in mouse model in highly T cell infiltrated organs, including the spleen, lymph nodes, and thymus compared to wild-type mice (59). Pre-clinically, Kristensen et al. investigated ^89^Zr-DFO-CD4, a radiolabeled rat anti-mouse F(ab)‘2 fragment. Evaluation of the radiopharmaceutical in numerous immunogenically “cold” and “hot” syngeneic tumor models treated with Sym021, an anti-PD-1 antibody, found that maximum tumor-to-heart values at the start of treatment were significantly able to predict response to therapy with immunologically cold tumors, with those that were poorly immune infiltrated being non-responders and those that were immunology hot, or highly tumor infiltrated, being responders to immunotherapy (60). This study demonstrated that CD4-specific radiopharmaceuticals could have utility in monitoring response to immunotherapy as well as identifying tumors that are already infiltrated with T cells, or immunologically “hot,” and thus are more likely to respond to immunotherapy-based treatments. However, like CD3 monitoring, because CD4 is expressed on both Tregs and CD4 helper T cells, its utility in stratifying responders and non-responders may be limited due to a lack of specificity for anti-tumor T cells.

#### 4.1.3 CD8 radiopharmaceuticals

Although radiopharmaceuticals targeting T cell lineage markers such as CD3 and CD4 have shown promise pre-clinically, significant progress has also been made in developing radiopharmaceuticals that target the T cell lineage protein CD8 both pre-clinically and in early-phase clinical trials. The presence of PD-L1/PD-1 expression in the tumor micro-environment is often not sufficient to confer ICI-mediated response, as CD8 T cells, or cytotoxic T cells, must also be present within the tumor immune microenvironment. CD8 T cells, after recognition of tumor-specific antigens, work through multiple mechanisms to confer anti-tumor activity, including the release of cytokines, granzyme B, and perforin or by engaging death receptor ligands (61) (Figure 2). In addition to providing a real-time readout of CD8 T cells at the tumor site, CD8-specific T cell-based PET imaging agents may also be able to capture systemic changes in CD8 T cell localization in response to ICI therapy.

Pre-clinically, Tavare et al. developed two anti-CD8 specific antibody fragments and labeled them with Copper-64. They demonstrated the specificity of their CD8 radiopharmaceuticals by comparing biodistribution and organ-specific uptake in WT B/6 mice compared to antigen-negative C3H mice and immunodeficient NOD SCID mice. In mice that expressed the antigen, they observed significant radiopharmaceutical uptake in the spleen, lymph nodes, and liver and high immune infiltrated organs, and in antigen-deficient mice or immunodeficient mice, they showed a significant decrease in radiopharmaceutical uptake in the spleen and lymph nodes, demonstrating specificity (62).

Subsequently, Tavare et al. developed an anti-CD8 cys-diabody (169 cDb) and labeled it with Zirconium-89, ^89^Zr-malDFO-169 cDb. They then utilized this radiopharmaceutical to monitor the response of mice harboring CT26 tumors to anti-CD137 agonist therapy and anti-PD-L1 treatment. Mice treated with anti-CD137 showed response to therapy as well as significantly increased uptake of the radiopharmaceutical in the tumors and lymph nodes of treated mice compared to untreated mice, and subsequent histologic analysis demonstrated an increase in CD8 T cells within treated tumors (63). In addition, when mice harboring CT26 tumors were treated with anti-PD-L1 therapy, responders showed significantly higher tumor uptake of ^89^Zr-malDFO-169 cDb compared to non-responders. However, compared to anti-CD137 therapy, there was not a similar increase in uptake in inguinal lymph nodes (63). These results demonstrated pre-clinically that CD8-targeted PET imaging agents can help predict immunotherapy response and that this correlates with increased CD8 T cell infiltration in the tumor compartment. The heterogenous amount of radiopharmaceutical uptake in the lymph nodes of mice treated with different immunotherapies points to how PET radiopharmaceuticals can also help to illuminate different systemic and spatial responses to immunotherapy.

In addition to successful pre-clinical testing, CD8-targeted PET imaging agents have also been shown to be effective predictors of immunotherapy response in several early-phase clinical trials. Pandit-Taskar et al. investigated a CD8-targeted minibody, ^89^Zr-IAB22M2, in 6 patients with hepatocellular carcinoma (HCC), lung cancer, and melanoma (64). Radiopharmaceutical uptake was observed at tumors, metastatic sites, and non-malignant lymph nodes. In addition, they observed the highest uptake in the spleen, liver, and bone marrow. ^18^F-FDG uptake at tumor sites did not always correlate with ^89^Zr-IAB22M2 uptake, potentially indicating that there was variability in the immunologic activity of distinct metastatic sites (64). In a subsequent larger study utilizing the same CD8-targeted PET imaging agent, Farewell et al. evaluated ^89^Zr-IAB22M2 uptake in 15 patients with melanoma, NSCLC, and HCC, half of whom were undergoing immunotherapy treatment. They similarly showed high radiopharmaceutical uptake in lymph nodes as well as heterogenous uptake at tumor sites (65). Although the study was not powered to evaluate if radiopharmaceutical uptake could be indicative of response to immunotherapy, they did find that in 3 patients, there was some correlation between higher radiopharmaceutical uptake at lymph nodes, nodal metastases, or metastatic sites and positive responses to immunotherapy treatment months later (65).

**Figure 3:**
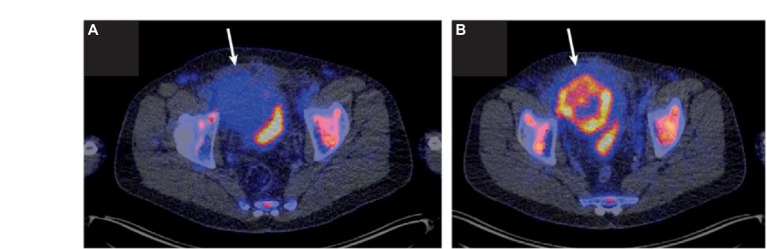
CD8 *in vivo* imaging. Imaging with CD8 radiotracer ^89^ZED88082A demonstrated heterogeneous tumor uptake and increased tumor uptake in response to immunotherapy. This imaging shows an initial minor uptake of ^89^ZED88082A in a dMMR urothelial cancer **(A)**, which increased after immunotherapy treatment **(B)**. (Adapted from (66) under the Creative Commons Attribution 4.0 International License). This figure was reproduced from Kist de Ruijter et al. (66) and licensed under the Creative Commons Attribution 4.0 International License.

Ruijter et al. evaluated a CD8-targeted one-armed antibody ^89^ZED88082A PET agent in 38 patients with a variety of cancers, including colorectal, cervical, melanoma, esophageal, HCC, ovarian, NSCLC, and TNBC, who were imaged 30 days before and 30 days after ICI treatment (66). In addition to demonstrating heterogenous tumor, lymph node, non-malignant lymph node, spleen, bone marrow, liver, and gut uptake, they also visualized distinct patterns of uptake with some tumors demonstrating uptake only around the tumor edges, indicating a potential invasive margin of T cells which increased in response to therapy (66) (Figure 3). Tumors with high MSI phenotype or those with an inflammatory phenotype identified by histology had high radiopharmaceutical uptake and CD8 expression by histology, which correlated with radiopharmaceutical uptake. They also observed higher radiopharmaceutical uptake at sites where patients experienced irAEs (66). Initial higher radiopharmaceutical uptake at tumor sites was significantly higher in patients with better overall response to ICI therapy; however, by some criteria, such as overall response by RECIST criteria, there were positive trends that did not quite reach statistical significance (66). These results demonstrate that CD8 radiopharmaceuticals can be utilized as potential effective agents *in vivo* to evaluate response to immunotherapy.

T cells play an essential role in the response to ICI therapy. Monitoring of T cell lineage markers has demonstrated some success in predicting response to ICI therapy both pre-clinically and clinically. Notably, markers such as CD3 that have broad expression on numerous types of T cells, including helper, Treg, and cytotoxic T cells, have been less effective at monitoring ICI response as they are unable to distinguish between an anti-tumor and pro-tumor T cell milieu. Similarly, although in a pre-clinical study, a CD4 PET imaging agent was able to predict response to immunotherapy, CD4 monitoring might have similar limitations as CD3 monitoring since CD4 is expressed on both T helper cells and T regs. CD8 PET imaging agents have made the most progress in monitoring response to ICI and have been tested in a variety of early-stage clinical trials. Clinically, CD8 PET imaging agents, which demonstrate higher initial tumor uptake, correlate to better overall response to ICI therapy. Notably, CD8 PET imaging correlates with CD8 infiltration by histology. In addition, CD8 imaging showed heterogeneous uptake in different tumors and along tumor margins, highlighting the complexity of CD8 T cell infiltration within tumors. Overall, CD8 monitoring, likely because of its specificity for cytotoxic T cells, one of the dominant effector cells in ICI therapy, has so far demonstrated the most utility in predicting response to ICI therapy clinically among lineage marker targeted radiopharmaceuticals (64–66).

## 5 T cell functional radiopharmaceuticals

As part of the multistep process of ICI therapy response, T cells must first infiltrate the tumor microenvironment. Once T cells are present, they must also exert cytotoxic activity. Thus, radiopharmaceuticals targeting T cell activity have been in development as they may demonstrate that T cells recruited to the tumor microenvironment are both present and effective.

### 5.1 Granzyme B

**Figure 4:**
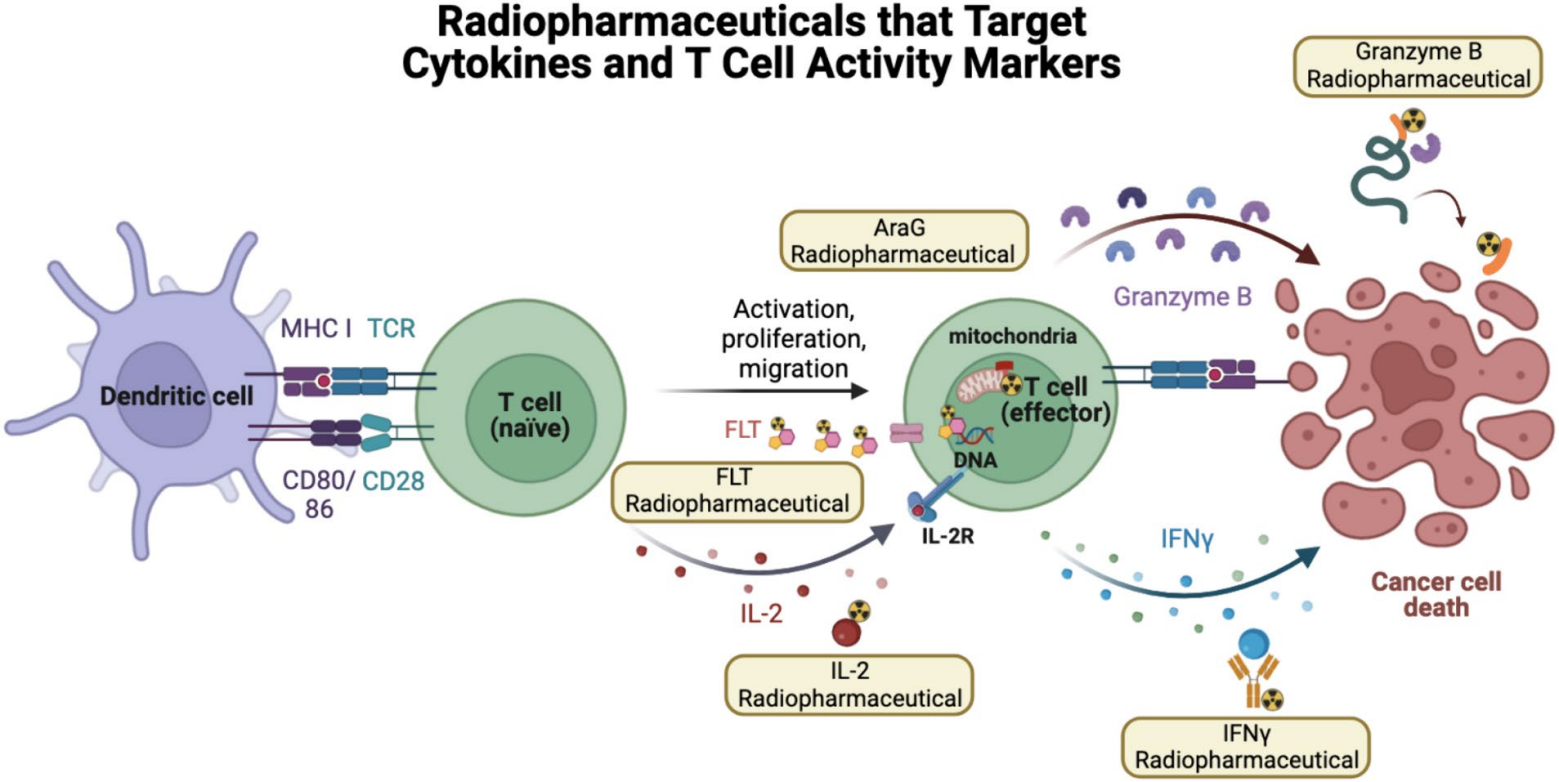
Radiopharmaceuticals that target cytokines and T cell activity markers. In order to monitor ICI response, another strategy has been to monitor markers of T cell activation or activity, such as IL-2, IFNy, Granzyme B, FLT, and AraG. Created using Biorender.com.

One mechanism of T cell-mediated cell killing is the release of the serine-protease granzyme B (Figure 4). Thus, radiopharmaceuticals that monitor the activity of granzyme B have the potential to monitor active T cell-mediated anti-tumor activity in real-time. Pre-clinically, Larimer et al. developed a granzyme B radiopharmaceutical GZP that could be cleaved by murine granzyme B and radiolabeled with Gadolidium-68. They then evaluated its uptake in mice harboring CT26 tumors that were treated with anti-PD-1 and anti-CTLA-4. Although Western blot analysis of CD8 or CD3 expression, or T cell infiltration, of treated and untreated tumors were the same, tumors that were treated with ICI had higher granzyme B expression by IHC, indicating activation of this T cell-mediated cell killing response. Furthermore, ^68^Ga-NOTA-GZP radiopharmaceutical uptake in tumors was able to significantly stratify CT26 tumor-bearing mice into responders and non-responders to immunotherapy. They also found that in biopsy samples of 9 melanoma patients treated with anti-PD-1 therapy, ICI responders had higher overall granzyme B expression by IHC. These results help to demonstrate that T cell activity markers may be effective predictors of systemic immune activation by ICIs and therapy response (67).

Goggi et al. evaluated another pre-clinical granzyme B-targeted PET imaging agent, [^18^F]AlF-mNOTA-GZP, in mice harboring HEPA 1–6 liver tumors treated with either anti-CTLA-4 or anti-PD-L1. Similarly, [^18^F]AlF-mNOTA-GZP imaging showed higher tumor uptake in mice that responded to immunotherapy treatment. Follow-up studies by flow cytometry demonstrated that therapy-responsive tumors had higher infiltration of NK cells (68). Using the same radiopharmaceutical, Hartimath et al. showed a similar ability to stratify mice harboring CT26 tumors who received both anti-PD-1 therapy and intratumor CpG-ODN injections, with CD8 T cells being the predominant correlate of response to therapy (69).

**Figure 5:**
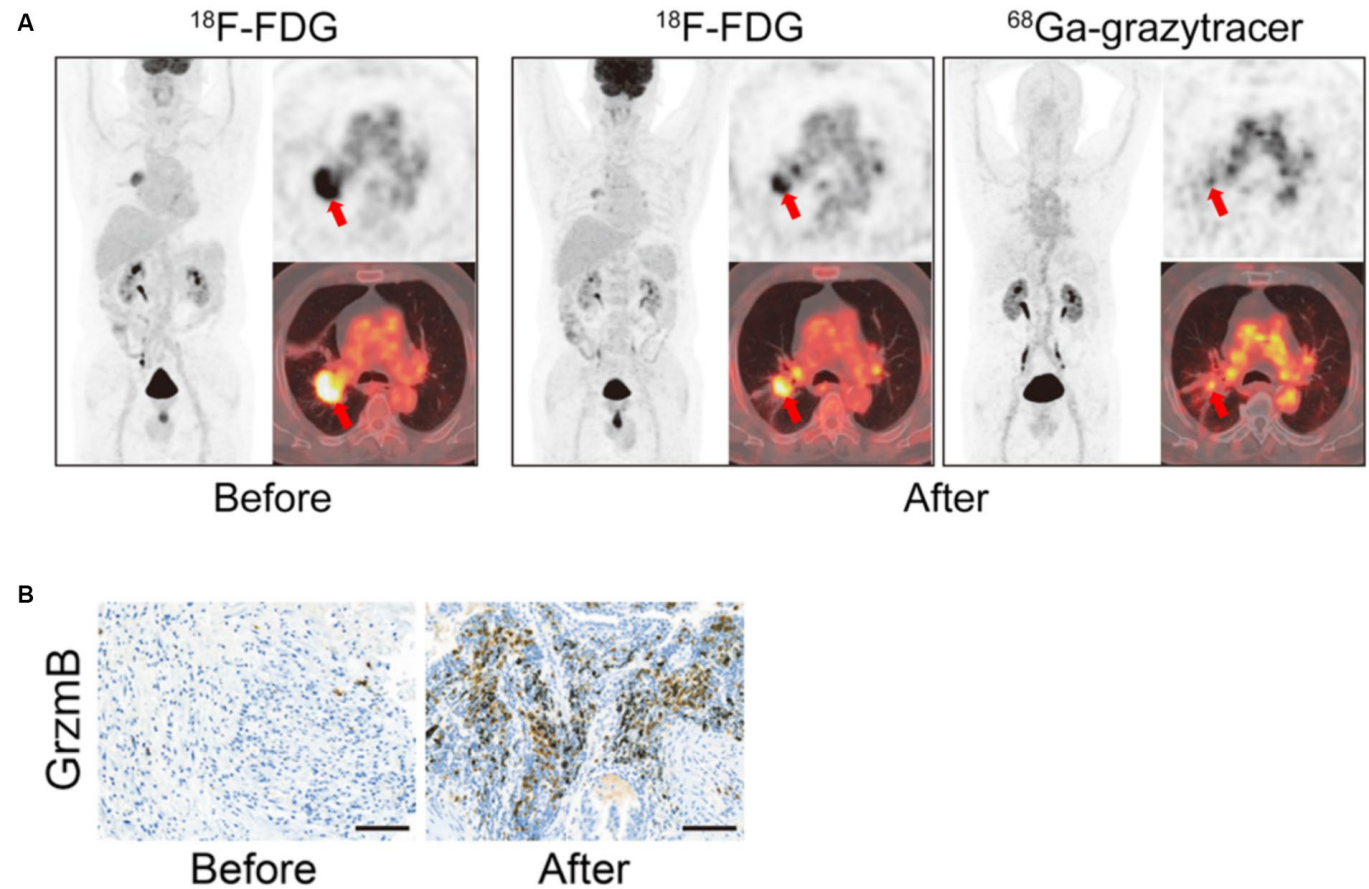
Granzyme B *in vivo* Imaging. Example of ^68^Ga-Granzyme imaging in a patient with lung adenocarcinoma before and after treatment with chemotherapy and anti-PD-1 demonstrates increased overall positive treatment response as demonstrated by the ^18^F-FDG images **(A)**. In addition, there was high tumor uptake of ^68^Ga-Granzyme uptake after treatment, and subsequent IHC staining of granzyme B in the tumor demonstrated increased granzyme B after treatment **(A,B)**. (Adapted from (70) under the Creative Commons Attribution 4.0 International License). This figure was reproduced from Zhou et al. (70) and licensed under the Creative Commons Attribution 4.0 International License.

Zhou et al. developed ^68^Ga-grazytracer, a 1,2,3-triazole-based non-aldehyde granzyme inhibitor, an adaptation of the GZP radiopharmaceutical. In pre-clinical models, ^68^Ga-grazytracer showed higher tumor-specific uptake and greater stability than ^68^Ga-NOTA-GZP. In addition, ^68^Ga -grazytracer imaging was able to stratify mice bearing MC38 tumors that responded or did not respond to anti-PD-1 therapy. In addition, they also were able to utilize ^68^Ga-grazytracer to effectively distinguish tumor pseudo progression from true tumor progression and identify early T cell infiltration. Finally, in a pilot clinical study, 5 patients with stage III and IV lung cancer and melanoma were imaged with both FDG and ^68^Ga-grazytracer a week after completing an immunotherapy treatment. Patients who had stable or partial response had higher overall tumor uptake of ^68^Ga-grazytracer compared to non-responders (70) (Figure 5). In this small case study, they identified a patient with lower ^68^Ga-grazytracer uptake and high PD-L1 expression by histology that progressed on anti-PD-L1 treatment, indicating that more than just expression of the immune check point target is necessary to confer therapeutic efficacy.

In pre-clinical studies, granzyme-B PET imaging agents have been successful in distinguishing tumors that respond and do not respond to immunotherapy treatment and have demonstrated promising correlative effects along the same lines in a small clinical pilot study. What is notable from these early-stage studies is that identification of just T cell infiltration or the presence of an immunotherapy target, such as PD-L1, may be helpful co-correlates of the potential for response to immunotherapy treatment but might not be sufficient as the T cells in the tumor milieu may not potentiate anti-tumor activity in response to immunotherapy activation. Measuring granzyme-B activity allows for a readout of activated T cells instead of just T cell presence within the tumor immune microenvironment.

### 5.2 T cell metabolism

#### 5.2.1 [^18^F]F-AraG

Another strategy to track T cell activation is to monitor T cell metabolism. An analog of arabinosyl guanine, AraG, is a compound that plays a role in the increased mitochondrial DNA synthesis that occurs when T cells become more metabolically active. AraG enters T cells and is phosphorylated by deoxyguanosine kinase. This kinase is essential for mitochondrial DNA synthesis. In addition, AraG has been shown to be uniquely selective for T cell-specific upregulation of mitochondrial DNA synthesis. As a result, radiolabeled AraG, [^18^F]F-AraG, has shown utility in monitoring T cell-specific activation and activity (71).

Ronald et al. evaluated [^18^F]F-AraG pre-clinically in a model of graft vs. host disease (GvHD). In GvHD mice, following bone marrow transplant, activated T cells accumulate in lymph nodes and the spleen. Subsequent imaging with [^18^F]F-AraG demonstrated specific uptake in cervical lymph nodes compared to controls, demonstrating the specificity of this radiopharmaceutical for activated T cells (72).

Levi et al. evaluated [^18^F]F-AraG pre-clinically in mice bearing MC38 tumors that were treated with anti-PD-1 therapy. Mice that responded to anti-PD-1 therapy had significantly higher uptake of ^18^F-AraG both at the tumor site and tumor-draining lymph nodes compared to mice that were non-responders. Subsequent FACs analysis revealed that there was no difference in the number of CD8+ and CD4+ T cells between responding and non-responding mice, indicating that [^18^F]F-AraG provides a true readout of the changes in T cell activation states in response to immunotherapy (73). These results also demonstrate that [^18^F]F-AraG can be used to longitudinally monitor and predict response to therapy.

In a subsequent pre-clinical study, Levi et al. utilized [^18^F]F-AraG to monitor CD8+ T cell activation in response to chemotherapy treatment (74). First, they evaluated [^18^F]F-AraG uptake in treatment naïve mice harboring varying syngeneic tumors, including MC38, CT26, LLC, A9F1, 4 T1, and B16F10 known to have different tumor immune infiltration. They observed heterogeneity in lymph node and tumor uptake of [^18^F]F-AraG between tumor types and within mice harboring the same tumor type. Subsequent FAC analysis of the tumor immune infiltrate demonstrated that [^18^F]F-AraG uptake specifically correlated with CD8+ PD-1+ cells. Subsequently, they investigated [^18^F]F-AraG uptake in mice harboring MC38 and A9F1 tumors undergoing two different chemotherapeutic treatments, one known to be immune priming and another not. [^18^F]F-AraG signal was significantly increased in tumors and lymph nodes of mice, in which chemotherapy treatment led to an immune priming response (74).

#### 5.2.2 ^18^F-FLT

Another method to measure cellular activity is FLT PET imaging. This radiopharmaceutical utilizes fluorine-18 labeled 3′-deoxy-3′-fluorothymidine (FLT). FLT is taken up by proliferating cells, phosphorylated by thymidine kinase, and then incorporated into DNA and can thus act as a marker of cellular proliferation (75). As such, FLT PET imaging has been explored as both a marker of tumor and immune cell proliferation (76). Ribas et al. evaluated ^18^F-FLT in 10 patients with melanoma undergoing anti-CTLA-4 therapy. Although uptake of ^18^F-FLT at tumor sites did not correlate with response to anti-CTLA-4 therapy, they did show a correlation between increased spleen uptake and overall increased response to therapy (77). In contrast, Scarpelli et al. evaluated ^18^F-FLT in 17 patients with metastatic prostate cancer treated with the pTVG-HP vaccine and pembrolizumab. In their study, high tumor uptake of ^18^F-FLT at baseline and spleen uptake was predictive of shorter overall progression-free survival (78). Yeh et al. evaluated ^18^F-FLT PET imaging in 5 patients with metastatic melanoma undergoing pembrolizumab treatment. Although they did not show significant effects, lower initial uptake of ^18^F-FLT at tumor sites and higher bone marrow-to-liver ratios were correlated with positive therapeutic responses (79).

Monitoring immune cell activity can act as an alternative marker for immune cell activation in response to immunotherapy. Although ^18^F-FLT has been evaluated as a potential clinical marker of immunotherapy response, mainly in melanoma patients, its lack of specificity for immune cells compared to other proliferating cells makes it a challenging biomarker. This has also led to clinical results that have discordant response patterns, with sometimes high radiopharmaceutical uptake or low radiopharmaceutical uptake being indicative of response to therapy (78, 79). In contrast, [^18^F]F-AraG imaging offers a more specific signal tailored to proliferating T cells as a surrogate marker of T cell activation. Pre-clinically, [^18^F]F-AraG imaging has shown success in discriminating between tumors that are responding and those that are not responding to immunotherapy. Notably, these pre-clinical studies have shown that in tumors with the same amount of CD4+ and CD8+ T cells, those with higher ^18^F-AraG tumor uptake were more likely to respond to immunotherapy. These results indicate that [^18^F]F-AraG PET imaging can help delineate beyond just T cell infiltration in the tumor microenvironment and also elucidate if those T cells are active. Although the results of [^18^F]F-AraG, detailed here, are all in pre-clinical models, [^18^F]F-AraG is actively being investigated as a biomarker for immunotherapy response in several ongoing clinical trials (NCT05701176, NCT05157659, NCT04260256, NCT04726215, NCT05096234, and NCT03142204).

### 5.3 Cytokines

#### 5.3.1 Il-2

Another strategy to monitor the T cell activation state is to develop radiopharmaceuticals that detect cytokine secretion. IL-2, when bound to the IL-2R on T cells, leads to T cell activation and proliferation and can act as a marker of T cell activity. Donk et al. evaluated [^18^F]FB-IL2, an IL-2 cytokine-based radiopharmaceutical (80) (Figure 4). In their study of ICI naïve patients with metastatic melanoma, they were able to visualize tumor sites but observed low radiopharmaceutical uptake. They also observed high uptake in the bone marrow and spleen; however, they did not observe any correlation between radiopharmaceutical uptake and ICI response (80).

#### 5.3.2 IFNγ

IFNγ is another cytokine that when secreted by T cells, directly participates in signaling involved in T cell-mediated killing (Figure 4). However, IFNγ can also lead to the upregulation of PD-L1. Pre-clinically, Gibson et al. radiolabeled an anti-IFNγ antibody with Zirconium-89. ^89^Zr-anti-IFNγ radiopharmaceutical uptake was higher in mice bearing TUBO tumors that received treatment with HER2/neu vaccinations and was more effective at distinguishing vaccinated compared to unvaccinated mice when compared to CD3 targeted PET imaging. In addition, higher initial ^89^Zr-anti-IFNγ uptake was significantly predictive of response to tumor vaccination and could be used to stratify responders and non-responders. However, in a model of T cell exhaustion, ^89^Zr-anti-IFNγ tumor uptake was no different from mice imaged with isotype control. These results indicate that ^89^Zr-anti-IFNγ PET imaging could be utilized as a marker of T cell activity as it is able to distinguish between T cell responses to therapy in activated T cells but shows little uptake when T cells are exhausted and unlikely to confer therapeutic effects when stimulated (81).

Cytokine monitoring can offer another methodology to distinguish activated T cells in the tumor microenvironment in response to immunotherapy treatment. Although limited to a few pre-clinical studies, IFNγ appears to be effective in stratifying immunotherapy responders and non-responders. It is unclear why IL-2R monitoring was less effective; however, it may be that the PET imaging agent was outcompeted by IL-2 itself in the tumor milieu.

## 6 Future directions

This review has focused mainly on radiopharmaceuticals that monitor T cell immunotherapy targets, T cell tumor infiltration, or markers of T cell activity because T cells play a dominant role in mediating immunotherapy response. However, the tumor immune microenvironment is a complex milieu that can contain both anti-tumor and pro-tumor immune cells of which some might actively impede T cell activation. In addition, other immune cells and non-immune cells in the tumor microenvironment might also play a role in driving an immunotherapy-driven therapeutic response.

As such, other pre-clinical radiopharmaceuticals have focused on tracking other cells in the tumor immune microenvironment, such as myeloid cells, macrophages, and even non-immune cells that can contribute to ICI resistance, such as fibroblasts (82). For example, progress has been made to detect and monitor CD11b, a tumor-associated myeloid marker, CD206, which is highly expressed in M2 macrophages, and TSPO, a mitochondrial receptor upregulated in activated macrophages via novel PET imaging agents (83–86). These non-T cell-focused PET imaging agents have significant promise in monitoring real-time changes of other factors within the tumor immune microenvironment.

## 7 Discussion and conclusion

Unlike a static biopsy, PET imaging can provide real-time, systemic information regarding ICI target expression and immune cell activity at multiple time points in response to ICI treatment. While there are many promising T cell-targeted PET imaging agents, the majority are still in the early stages of development and have yet to be tested in larger clinical trials (Table 1). The PET imaging agents ^89^Zr-pembrolizumab (PD-1), ^89^Zr-atezoluzumab (PD-L1), and ^89^ZED88082A (CD8) have demonstrated the most promising clinical predictive responses to ICI therapy in small scale first in human or phase 1/2 trials. Using different SUVmax cutoffs of overall tumor uptake, each of these radiopharmaceuticals demonstrated, during a 24–48 month follow-up period, that patients with greater initial tumor uptake had significantly greater overall survival, progression-free survival, or stable disease compared to patients that fell below the cutoff, as measured by hazard ratios. Among these three agents, ^89^Zr-atezoluzumab demonstrated the most significant hazard ratios. In addition, ^89^Zr-atezoluzumab performed better than the IHC PD-L1 markers SP2963 and SP142. Notably, SP142 was unable to statistically distinguish patients who achieved progression-free or overall survival in the same cohort. However, how these imaging agents perform on an individual basis in an ROC analysis has yet to be evaluated. In addition, clinical studies that evaluated these agents were small, with the largest cohort involving 34 patients, used summation statistics, grouped overall, progression-free, and stable diseases together, and relied on cohorts that combined patients with several tumor types. In addition, further validation of these imaging agents is also needed. Only ^89^Zr-atezoluzumab (PD-L1) and ^89^ZED88082A (CD8) were validated via autoradiography and showed that high tissue uptake of each agent significantly correlated with relevant marker IHC staining of the same tissue. Although these clinical studies are small, these three imaging agents have demonstrated the most promise in predicting response to ICI therapy clinically. However, larger studies are needed to further determine how these metrics perform based on tumor type and immunotherapy-based treatment.

In addition, clinically and pre-clinically, PET imaging agents were often more effective than IHC at predicting response to immunotherapy, and radiopharmaceutical uptake did not always correlate with IHC staining. These findings further support the utility of PET in capturing whole-body heterogeneity of target expression compared to single-site information that can be provided by a biopsy. In addition, unlike a static biopsy, PET imaging can be used to monitor response to therapy at multiple time points during treatment. Further studies to understand the changes in radiopharmaceutical uptake in tumors at multiple timepoints during ICI therapy will help to provide insights into how these markers are modulated throughout treatment and could potentially uncover early indicators of impending resistance to treatment.

Notably, all clinically tested radiopharmaceuticals also demonstrated heterogeneous tumor uptake at tumor sites and metastases, which did not always correlate with FDG PET. These observations further demonstrate that T cell-targeted PET radiopharmaceuticals provide improved characterization of the heterogeneity of the tumor immune infiltrate as compared to FDG PET. In addition, this also demonstrates that the immune infiltrate at metastases may not be uniform, indicating that not all metastases may respond in the same way to ICI therapy.

In addition to demonstrating uptake in primary tumors and metastases, many of the clinically tested radiopharmaceuticals demonstrated uptake in benign areas such as non-malignant lymph nodes, Waldyer’s ring, bone marrow, spleen, sites of inflammation, and at sites where patients developed irAEs. These findings indicate that these radiopharmaceuticals may also be able to provide a systemic readout of the overall immune activation state of the patient. Further investigation into whether this global inflamed state leads to higher response rates to ICI warrants further evaluation.

Response to ICI therapy requires multiple stages, including available therapeutic targets, infiltration of T cells, and activation of those T cells. Monitoring target expression and T cell infiltration has already demonstrated efficacy in predicting immunotherapy response clinically. However, T cell infiltration alone may not be sufficient to confer immunotherapy response as many other factors may impede T cell-mediated anti-tumor efficacy. Although still in the preliminary stages, PET imaging agents that monitor T cell activation are promising new strategies to predict immunotherapy response that warrant further investigation.

Herein, we have reviewed pre-clinical and clinical studies of T cell-based PET imaging agents. Excitingly, many of these radiopharmaceuticals, in both clinical and pre-clinical studies, have demonstrated utility in predicating response to ICI treatment. However, these studies remain small, and only a few radiopharmaceuticals have been tested in phase 1 or 2 clinical trials. Larger clinical trials are needed to evaluate these agents. Nonetheless, T cell-based PET imaging agents show significant promise as a potential real-time biomarker that could be used to stratify patients who are or are not responding to ICI treatment.

## Author contributions

SG researched and wrote the initial manuscript. All authors contributed to the article and approved the submitted version.

## Glossary

Adnectins: proteins designed based on the fibronectin III domain with high affinity for proteins of interest
CAR-T: chimeric antigen T cell receptor therapy
CpG: a cysteine and guanine separated by a phosphate that activates toll-like receptor 9
CTLA-4: cytotoxic T lymphocyte associate antigen
cys-diabody: two Fv fragments connected by a peptide linker
dMMR: deficient mismatch repair
F(ab’)2 fragment: part of an antibody that contains two antigen-binding domains linked by disulfide bonds
FcγR: Fc gamma receptor
FDG: fluorodeoxyglucose
GvHD: graft versus host disease
HCC: hepatocellular carcinoma
ICI: immune checkpoint inhibitors
IFNγ: interferon gamma
IHC: immunohistochemistry
IL-2: interleukin 2
irAE: Immune-related adverse effects
iRECIST: immune response evaluation criteria in solid tumors
LAG-3: lymphocyte activating gene 3
MDSC: Myeloid-derived suppressor cells
MHC: major histocompatibility complex
MSI: microsatellite instability
NGS: next generation sequence
NK: natural killer cells
NSCLC: Non-squamous cell lung cancer
OX40: a type of tumor necrosis factor receptor
PCR: polymerase chain reaction
PD-1: programmed cell death protein 1
PD-L1: programmed cell death ligand 1
PET: positron emission tomography
Probody: antibody that is cleaved and activated in the tissue of interest
TCR: T cell receptor
TIGIT: T cell immunoglobulin ITIM domain
TIL: tumor infiltrating lymphocyte
TMB: tumor mutational burden
TNBC: Triple-negative breast cancer
TNFa: tumor necrosis factor-alpha
Waldeyer’s ring: ring of lymphoid tissue around the throat
WES: whole exome sequencing
